# Utilization of Heritage Manifestations in a Reminiscence Programme (App) Directed at Older Adults with and Without Neurocognitive Disorder, Employing AI: A Pilot Study in Portugal and Spain

**DOI:** 10.1007/s10823-026-09581-0

**Published:** 2026-06-08

**Authors:** Natália Albino Pires, Pedro Machado dos Santos, Sara Doménech, Àngela Nebot, Francisco Mugica, Anass Benali Bendahmane, Xènia Porta

**Affiliations:** 1 inED - Center for Research and Innovation in Education, Polytechnic University of Coimbra, Coimbra, Portugal; 2 UNESCO Chair in Intangible Heritage and Traditional Know-how, Évora, Portugal; 3 CINTESIS – Center for Health Technology and Services Research, Oporto, Portugal; 4 Fundació Salut i Envelliment, Universitat Autònoma de Barcelona, Barcelona, Spain; 5 Soft Computing Research Group at Intelligent Data Science and Artificial Intelligence Research Center, Universitat Politènica de Catalunya, Barcelona, Spain; 6 Universitat Oberta de Catalunya, Barcelona, Spain; 7 Escola Superior de Educação, Instituto Politécnico de Coimbra, Coimbra, Portugal

**Keywords:** Traditional Oral Literature, Traditional Dance, Intangible Heritage, Reminiscence Therapy, LONG-REMI App, Artificial Intelligence, Older Adults, Pilot Study

## Abstract

Cultural and intangible heritage has been part of human daily life since time immemorial, fulfils a function within the community and acts as an element of identity. Its potential as a stimulus in Reminiscence Therapy (RT), however, has been scarcely explored, despite well-documented evidence on the role of music, crafts or sports as facilitators of autobiographical recall. This pilot study had three operational objectives: (i) to design a reproducible protocol for selecting elements of intangible cultural heritage (traditional oral literature and traditional dances) suitable for personalised RT; (ii) to develop and field-test the first prototype of the LONG-REMI App, an AI-supported tablet application that delivers these stimuli in an individualised manner; and (iii) to examine the impact of those stimuli on memory recall, communication and interaction in older adults with and without neurocognitive disorder. Pilot, prospective, multicentre, mixed-methods study with a participatory observational component, conducted in six institutions (three Day Care Centres in Catalonia (Spain) and three centres in Portugal) between February 2021 and April 2022. Fifty-six participants aged ≥ 65 years were enrolled (*n* = 21 without cognitive decline, recruited from two centres; *n* = 35 with mild or moderate cognitive decline, recruited from four day-care centres). Mean age was 80.71 ± 6.82 years (range 66–94); 41 (73.21%) were women and 43 (76.78%) had a low educational level (including two illiterate participants). Cognitive status was classified using Reisberg’s Global Deterioration Scale (GDS 3 and 4) and the Mini-Mental State Examination (MMSE; group with cognitive decline: 22.26 ± 5.90, range 7–30). Each participant attended four weekly individual 45-minute sessions on Android tablets. The App database contained more than 500 multisensory stimuli organised in two difficulty modalities (low / high). Data were collected through the Visual Analogue Scale (VAS, 1–10) for usability and satisfaction; the Spanish-adapted Computer System Usability Questionnaire (CSUQ, 7-point Likert); and the Spanish-validated PANAS, administered before and at the end of the intervention. In addition, a structured observation grid (memory recall, verbal and non-verbal interaction), audio-video recording and facilitators’ field notes supported the qualitative arm. Content validation of the App database was carried out by the multidisciplinary research team itself, through consensus during project meetings; a formal Delphi with an external panel is foreseen for the planned randomised trial. Stimuli combining rhyme and music (songbook items and ballads) and stimuli supported by video produced the highest frequency of recall episodes and of communication / interaction events. Brief audio-only stimuli (proverbs and nursery songs without video) elicited fewer recall events and were associated with observed disengagement. Usability and satisfaction were highly perceived in both groups: VAS usability 7.75 ± 1.88 and VAS satisfaction 8.38 ± 1.57 on a 1–10 scale; the CSUQ overall score was 6.95 ± 0.22 in the group without cognitive decline and 5.44 ± 1.09 in the group with cognitive decline (1–7 Likert). PANAS scores showed a statistically significant shift in positive affect from pre- to post-intervention (28.86 ± 8.88 vs. 36.70 ± 9.43; Z = − 4.18; *p* < 0.001), while no significant change was observed in negative affect (12.09 ± 3.19 vs. 11.89 ± 3.00; *p* = 0.688). Participants completed on average 3.59 ± 0.40 of the four planned sessions. Traditional oral literature and traditional dance, when delivered through an AI-supported App, can act as effective triggers for reminiscence in older adults with and without cognitive decline. The study contributes (a) a replicable protocol for cross-cultural selection of intangible heritage stimuli, (b) preliminary evidence on which formats (audio + video, rhyme + music) are most effective, and (c) an open methodological framework for future randomised, longitudinal trials.

## Introduction

Human development and social heritage are largely based on different practices, expressions, traditions, knowledge and techniques transmitted by communities from generation to generation: intangible heritage (*UNESCO – Intangible Cultural Heritage*, n.d.). Traditional and oral literature, along with music, dances, rituals and games, are examples of heritage that have been part of human daily life since time immemorial (Huizinga, 1951; Niles, 1999); they fulfil a function within the group (Espírito Santo, 2005; Finnegan, 1992; Paz Frayre et al., 2018; Utley, 1958) and act as identity elements (Bigand & Tillmann, 2020; Halbwachs, 1968; Oppong, 2013; Ricoeur, 2007). As an intrinsic element of the social life of their bearers (Bauman, 1986, 2000; Niles, 1999), all performative acts in which they are expressed maintain a close relationship with human cognition – through the storage, processing and representation of information in the brain (Connell, 2019; Grimaldi, 2012; Pinker, 1997) – and an equally important relationship with the emotional dimension, essential for affective bonds and mnemonic processes on which both semantic and episodic memory are interdependent (Damásio, 2010, 2011, 2022; Davis & Yee, 2021; Riegel et al., 2016; Robertson, 2002).

Today, the planning of strategies to safeguard cultural and intangible heritage and to optimise its potential within sustainable development (*UNESCO – Intangible Cultural Heritage*, n.d.) places older adults in a position of particular prominence (Themudo Barata et al., 2021), while reiterating warnings about the sociocultural and demographic changes currently under way (*United Nations*, n.d.; *World Report on Ageing and Health*, n.d.).

While the value of collective memory is widely recognised, we are increasingly faced with a growing number of older adults who experience losses or changes in their own memory. Cognitive changes are one of the main factors responsible for altering daily-living habits and reducing quality of life in older age (Alzheimer Europe, 2019), and there is a consensus that mental inactivity may not only increase the risk of neurocognitive decline but also accelerate the loss of these faculties at advanced ages (e.g., Daviglus et al., 2010). Engaging older people in cognitive-stimulation programmes can slow this decline and improve their quality of life (Woods et al., 2006).

Among activity programmes aimed at older adults, Reminiscence Therapy (RT) has shown effective results (Woods et al., 2006, 2018) in improving the quality of life of people who experience mild or moderate cognitive decline (Reisberg et al., 1982). RT has proved to be a powerful rehabilitation technique in different neurocognitive disorders (e.g., Miguel-Hidalgo et al., 2022), as it focuses on the primary role of emotions (Damásio, 2010, 2011, 2022) for the development and reinforcement of identity, rather than only on memory deficits (e.g., Cuevas et al., 2020). Structured reminiscence involves using facilitative stimuli to evoke meaningful, personalised memories associated with participants’ past experiences (Woods et al., 2006, 2018). Oral literature and dance, which play a determining role in the construction and maintenance of group identity and in the preservation of collective and individual memory, can therefore be used as facilitating stimuli in RT sessions.

An innovative way to promote RT involves the use of assisted technological applications (Carós et al., 2020; Cuevas et al., 2020). The development of computer systems with adaptive capabilities (Lake et al., 2017) makes it possible to explore new intersections between traditional fields of knowledge. In the present project, Artificial Intelligence (AI) plays a specific and bounded role: the App uses an AI engine to (a) personalise the sequence of stimuli on the basis of the participant’s previous responses (preference learning), (b) automatically classify the participant’s reaction (engagement/disengagement) from the interaction with the touch screen, and (c) generate adaptive feedback for the facilitator. More specifically, as described in Nebot et al. (2022), the App combines two AI components: a face-tracking module based on the ML Kit SDK and TensorFlow Lite that computes a smile index in the range [0, 1] from the contours of the eyes and mouth, sampled once per second; and a reinforcement-learning algorithm that operates on a fully connected weighted graph for each heritage category (proverbs, nursery songs, songbook items, dances), updating the weight of each item according to the user’s smile index and explicit feedback. AI is therefore not used as a general-purpose tool but as an explicit component of the personalisation loop.

To date, on the basis of a systematic exploration of the literature performed in PubMed, Web of Science, Scopus and Google Scholar (search terms: ‘reminiscence therapy’ AND ‘app’ OR ‘intangible heritage’ OR ‘oral literature’ OR ‘traditional dance’; period 2010–2024), no technological application has been identified that delivers RT and simultaneously guides users through content related to intangible heritage — such as oral literature and traditional dance – based on the reaction and emotional assessment of users with or without neurocognitive disorders.

Although there is scientific evidence on diverse stimuli that facilitate RT – such as music (e.g., Cunningham et al., 2019; Mahendran et al.,^2017^), crafts (e.g., Pöllänen & Hirsimäki, 2014), the visual arts (e.g., Keating et al., 2020) or football (e.g., Coll-Planas et al., 2017) – certain elements of intangible heritage, in particular traditional dance and some genres of traditional literature (sung or recited, with greater or lesser formal openness), have been considerably less used in cognitive-stimulation programmes.

We therefore start from two assumptions, which are theoretically grounded in the model of emotion-driven encoding proposed by Damásio (2010, 2011) and in the cue-dependent retrieval framework (Riegel et al., 2016; Robertson, 2002): (i) the heritage elements proposed as stimuli for RT sessions correspond to images stored from emotionally significant contexts of the participants; and (ii) the heritage elements proposed as stimuli allow the connection between the participants’ past and present to be activated, intensifying the correspondence between memory and identity.

From these assumptions we derive three operational research questions: (RQ1) Is it feasible to construct a culturally equivalent, AI-supported reminiscence App from intangible-heritage repertoires available in two distinct linguistic regions (Catalonia and Portugal)? (RQ2) Among the heritage formats considered (proverbs, nursery songs, songbook, traditional dance), which formats elicit the greatest number of recall episodes, communication acts and interaction events? (RQ3) Are these patterns similar in older adults with and without neurocognitive decline?

In this study we present the methodological proposal for selecting and implementing the heritage collection included in the App and we reflect on the impact of using stimuli from traditional literature (sung or recited) and traditional dance, as heritage elements, in RT sessions with the support of AI, taking into account their interrelation with the polyconcept of memory (Cantarino & Pereira, 2004; Halbwachs & Díaz, 1995; Rábano Gutiérrez, 2021; Ricoeur, 2007) and with mnemonic processes (Damásio, 2011; Davis & Yee, 2021; Riegel et al., 2016; Robertson, 2002).

## Intangible Heritage in a Reminiscence Programme

In this work we seek to identify a systematic method that supports the resolution of a typical research problem – the selection of content for reminiscence programmes – through (i) data collection using various techniques, (ii) interpretation of the collected data and (iii) the formulation of conclusions about the research data.

This methodological approach largely coincides with the LONG-REMI research project (Doménech et al., 2023; Nebot et al., 2022), in that it provides a set of principles and ideas that informed the design of the present study. More specifically, the manuscript should be read as a methodological paper with an empirical pilot component: it presents (a) the protocol for content selection and validation and (b) preliminary observational evidence on the impact of the resulting App on memory recall, communication and interaction during RT sessions.

### Materials and Methods

#### Study Design

 This is a pilot, prospective, multicentre, mixed-methods study with a participatory observational component. The qualitative arm (dominant) consists of structured field observation and content analysis of session field notes and audio-video recordings; the quantitative arm (subordinate, exploratory) consists of pre-/post-session affect measurement (PANAS) and usability scoring (SUS). The two arms are integrated through methodological triangulation. The choice of a pilot mixed-methods design is justified by the exploratory nature of the research question (the use of intangible heritage as a stimulus in AI-supported RT had not been described before) and by the need to obtain both numerical indicators (suitable for sizing a future randomised trial) and rich qualitative descriptions of the participants’ engagement.

#### Ethical Considerations

The study was conducted in accordance with the ethical principles of the Declaration of Helsinki and was approved by the Ethics Committee for Animal and Human Experimentation (CEEAH) of the Universitat Autònoma de Barcelona, Spain (protocol code CEEAH 5380, approved on 18 December 2020) and by the Ethics Committee of the Polytechnic Institute of Coimbra (Comissão de Ética do Instituto Politécnico de Coimbra), Portugal (protocol code 104_CEPC2/2020, approved on 26 November 2020). Written informed consent was obtained from all participants prior to inclusion; for participants with cognitive decline assessed as moderate or above, informed consent was additionally signed by their legal representative, and the participant’s assent was sought at every session. Participation was voluntary and free of charge, and participants could withdraw at any moment without consequence. All audio-video recordings were stored on encrypted institutional servers and were accessible only to the research team. Personal data were processed in accordance with Regulation (EU) 2016/679 (GDPR), Portuguese Law 58/2019 and Spanish Organic Law 3/2018.

#### Sample

Recruitment was carried out through non-probabilistic convenience sampling in the six participating institutions. Inclusion criteria: aged 65 years or older; institutionalised in a participating centre; able to give informed consent (directly or through a legal representative); preserved sensory function compatible with audio-visual interaction (with or without correction). Participants were not required to be able to read and write. Exclusion criteria: severe mental, sensory, behavioural and/or cognitive alterations that could interfere with the intervention; expected discharge before the end of the four-session protocol (Table 1).

**Table 1:** Demographic and clinical characteristics of the participants (extracted from Doménech et al., 2023)

	Without cognitive decline (*n* = 21)	With cognitive decline (*n* = 35)
Age (years), M ± SD (range)	76.67 ± 6.55 (66–87)	81.76 ± 7.08 (68–94)
Sex — women, n (%)	14 (66.7%)	[Authors: confirm n]
Educational level — low, n (%)	8 (38.1%)	35 (100%)
Educational level — medium, n (%)	10 (47.6%)	0 (0%)
Educational level — high, n (%)	3 (14.3%)	0 (0%)
MMSE, M ± SD (range)	—	22.26 ± 5.90 (7–30)
GDS stage	1–2	3–4
Sessions completed, M ± SD (max = 4)	3.59 ± 0.40 (whole sample)	3.59 ± 0.40 (whole sample)

A total of 56 participants were enrolled: 21 from two centres for older adults without cognitive decline and 35 from four day-care centres for older adults with cognitive decline, distributed across Spain (Catalonia) and Portugal. The sample had a mean age of 80.71 ± 6.82 years (range 66–94); 41 (73.21%) were women, 43 (76.78%) had a low educational level (no formal studies or primary studies only) and two participants were illiterate. Cognitive status was classified by the clinical team of each centre using the Global Deterioration Scale (Reisberg et al., 1982; GDS 3 = ‘mild cognitive decline/mild stage of dementia’, GDS 4 = ‘moderate cognitive decline/moderate stage of dementia’) and the Mini-Mental State Examination (Folstein et al., 1975) administered to the cognitive-decline group, which scored a mean of 22.26 ± 5.90 (range 7–30). The demographic and clinical characteristics of the two groups are reported in Table 2.

**Table 2:** Methodological summary of the study

Methodological dimension	Description in this study
Type of study	Pilot, prospective, multicentre, mixed-methods study with a participatory observational component. The qualitative dimension (structured field observation) is dominant; the quantitative dimension is exploratory and supports the qualitative findings.
Research question	Can elements of intangible cultural heritage (traditional oral literature and traditional dances), delivered through an AI-supported App, function as effective stimuli to evoke autobiographical memories and promote communication and interaction in older adults with and without neurocognitive disorder?
Setting	Six institutions: three Day Care Centres in Catalonia (Spain) – Casal de Gent Gran del Baix Guinardó, Associació Nou Horitzó, Centre de dia Allegrare; and three centres in Portugal – Centro Social e Cultural de Ribamar, Santa Casa da Misericórdia da Amadora, Alzheimer Portugal/Delegação do Centro em Pombal.
Sample	Fifty-six participants ≥ 65 years (*n* = 21 without cognitive decline; *n* = 35 with mild or moderate cognitive decline). Mean age 80.71 ± 6.82 years (66–94); 41 women (73.21%); 43 with low educational level (76.78%), including 2 illiterate. Inclusion: aged ≥ 65; institutionalised in a participating centre; able to give informed consent (directly or through legal representative); ability to read and write was NOT required. Exclusion: severe mental, sensory, behavioural and/or cognitive alterations that could interfere with the intervention.
Cognitive screening	Cognitive status classified using Reisberg’s Global Deterioration Scale (Reisberg et al., 1982); GDS 3 = mild and GDS 4 = moderate cognitive decline. Mini-Mental State Examination (Folstein et al., 1975) administered to the cognitive-decline group: 22.26 ± 5.90 (range 7–30).
Recruitment	Non-probabilistic convenience sampling. Participants were invited by the clinical staff of each institution; those willing and meeting the inclusion criteria signed a written informed consent (in the case of moderate cognitive decline, also signed by a legal representative).
Intervention	Four weekly individual sessions of approximately 45 min per participant, delivered through the LONG-REMI App on Android tablets, with personal headphones. Average sessions completed: 3.59 ± 0.40. Each session followed a five-step structure (welcome and PANAS pre-test → free choice of category → presentation of stimuli with facilitator prompts → closing conversation → PANAS post-test).
Stimuli categories	Four categories of intangible heritage: traditional dances, songs (songbook), proverbs, and nursery songs (tongue twisters in the Catalan version). The App database contained more than 500 multisensory stimuli organised in two difficulty modalities (low/high).
Variables and instruments	Affect: PANAS (Spanish-validated version, Robles & Páez, 2003) administered pre and post intervention. Usability and satisfaction: Visual Analogue Scale (VAS, 1–10) and Spanish-adapted CSUQ (7-point Likert; Hedlefs et al., 2015). Engagement, communication and interaction: structured observation grid with operational definitions of memory recall, verbal interaction and non-verbal interaction.
Data analysis	Descriptive statistics for PANAS, VAS and CSUQ scores. Non-parametric Wilcoxon signed-rank test for paired pre-/post-intervention comparisons (significance set at *p* < 0.05). All quantitative analyses performed in IBM SPSS Statistics version 21. Thematic content analysis (Braun & Clarke, 2006) of session field notes and audio-video recordings of recall episodes. Triangulation between the three data sources.
Expert validation	Content validation by the multidisciplinary research team itself (gerontology, neuropsychology, ethnomusicology, oral-literature studies, computer science). Items were reviewed and selected by team consensus during project meetings; items considered culturally or developmentally inadequate were modified or excluded. A formal Delphi procedure with an external panel and quantified agreement thresholds is planned for the next phase of the project (randomised trial).
Ethics	Approved by the Ethics Committee for Animal and Human Experimentation of the Universitat Autònoma de Barcelona, Spain (CEEAH 5380, 18 December 2020) and by the Ethics Committee of the Polytechnic Institute of Coimbra, Portugal (104_CEPC2/2020, 26 November 2020). Conducted in accordance with the Declaration of Helsinki. Data processed in compliance with Regulation (EU) 2016/679 (GDPR), Portuguese Law 58/2019 and Spanish Organic Law 3/2018.
Funding	Co-financed by the Fundación General CSIC, within the project ‘Programa para una Sociedad Longeva’ (0551_PSL_6_E), Programme Interreg V-A Spain–Portugal (POCTEP) 2014–2020, of the European Regional Development Fund (FEDER).

#### The LONG-REMI App

LONG-REMI is the name of an App supported by AI which delivers RT based on manifestations of intangible heritage in a periodic, individualised manner, guided by a facilitator (therapist or caregiver) and adapted to the level of cognitive decline and the older adult’s preferences (Doménech et al., 2023; Nebot et al., 2022).

The technical characteristics of the App are described in Nebot et al. (2022); the results of its usability and its effect on positive and negative affect (PANAS) in a sample of subjects residing in Spain and Portugal are reported in Doménech et al. (2023).

LONG-REMI is also the name of a pilot study, financed by Fundación General CSIC as part of the project “Programa para una Sociedad Longeva” (0551_PSL_6_E), within the Programme Interreg VA Spain–Portugal (POCTEP) 2014–2020, of the European Regional Development Fund (ERDF).

#### Intervention Procedure

A first prospective and multicentre observational study was carried out in Spain and Portugal between February 2021 and April 2022, in five Day Care Centres (three in Spain, two in Portugal) and one Assisted Living Facility (in Portugal). Male and female participants over 65 years of age, with and without cognitive decline, were eligible for inclusion (Doménech et al., 2023). The intervention consisted of four weekly sessions of approximately 45 min, on the premises of the partner institutions, in a welcoming environment with capacity for simultaneous sessions of three to four users. The App database contained more than 500 multisensory stimuli organised in two difficulty modalities (low/high), which were dynamically allocated by the facilitator to each participant according to the participant’s GDS stage and educational level.

Each session followed a standard structure: (1) welcome and PANAS pre-test (≈ 5 min); (2) free choice of one of the four heritage categories on the App home screen – proverbs, nursery songs, songbook or dances (≈ 2 min); (3) presentation of stimuli and interaction with the App, with the facilitator prompting recall through a fixed set of open-ended questions about the learning context of each stimulus (≈ 30 min); (4) closing conversation in which the participant was invited to comment on the session and (5) PANAS post-test (≈ 8 min). The exact sequence of stimuli was personalised by the App based on the participant’s previous choices and reactions.

The App, described in Nebot et al. (2022), allows RT sessions to be carried out periodically and in an individualised manner, adapted to the participants’ level of engagement and their preferences. Each participant identified themselves with a username (“user X”) on one of the Android devices (tablets) on which the App was installed, allowing the same tablet to be used by the respective user in every session. All participants wore comfortable headphones, so that the stimuli presented to each participant did not interfere with those presented to the other participants. During the sessions, one of the researchers and a specialised professional, previously trained on the App, were always present and supervised every session.

The App interface allows the user to choose the category (proverbs, nursery songs, songbook or dances) they prefer. The application then presents a stimulus, followed by questions about its learning context. The participant can return to the initial menu whenever they wish to choose another category.

#### Variables, Operational Definitions and Data-Collection Instruments

The dependent variables were operationalised as follows:

- Memory recall episode: any verbal or non-verbal manifestation in which the participant spontaneously associates the presented stimulus with an autobiographical event, a person or a context external to the session. Coded as a binary event (present/absent) per stimulus and counted per session.
- Verbal interaction: any verbalisation directed at the facilitator, another participant or the App, exceeding three words, that is contingent on the stimulus. Counted per stimulus and per session.
- Non-verbal interaction: any observable motor response contingent on the stimulus (e.g., tapping the rhythm, reproducing dance steps, smiling at a familiar image). Counted per stimulus and per session.
- Affect: positive and negative affect measured with the Spanish-validated version of PANAS (Robles & Páez, 2003; Watson et al., 1988), composed of 20 items (10 positive, 10 negative) rated on a 5-point Likert scale, administered before and after the intervention.
- Usability and satisfaction: assessed with the Visual Analogue Scale (VAS, 1–10; Heller et al., 2016) at the end of the intervention, and complemented with the Spanish-adapted Computer System Usability Questionnaire (CSUQ; Hedlefs et al., 2015; Sauro & Lewis, 2012), 7-point Likert (1 = strongly disagree; 7 = strongly agree), comprising 16 items grouped into system use (items 1–6), information quality (items 7–12), interface quality (items 13–15) and overall estimation (item 16).

Data were collected with three instruments: (1) the PANAS, VAS and CSUQ questionnaires; (2) a structured observation grid built ad hoc and pre-tested in two pilot sessions, including the four behavioural categories above plus open-text fields for facilitator notes; (3) audio-video recording of all sessions, used as a verification source. In the present pilot, each session was observed by a single trained facilitator at each centre, who completed the structured observation grid in real time; the audio-video recordings were retained as a verification source for cross-checking specific recall episodes during the analysis phase. A formal inter-rater reliability index (Cohen’s κ) was therefore not computed at this stage, since the primary aim of the pilot was to establish the feasibility of the protocol and the App. Systematic double-coding by two independent observers and the calculation of κ are foreseen for the planned randomised trial.

#### Data Analysis

Quantitative variables (PANAS scores, VAS scores, CSUQ scores, frequency counts of recall, verbal and non-verbal interaction) were analysed using descriptive statistics (mean, standard deviation, median, interquartile range) and exploratory comparisons across stimulus categories using non-parametric tests (Wilcoxon signed-rank for paired pre-/post-session measures; Mann–Whitney U for between-group comparisons). Significance was set at α = 0.05. Analyses were performed in SPSS Statistics version 21 (IBM Corp.). Field notes and recall episode transcripts were submitted to thematic content analysis (Braun & Clarke, 2006), with codes derived both inductively and deductively. Methodological triangulation between the three data sources (observation grid, recordings, field notes) was used to enhance trustworthiness.

#### Content Validation by the Multidisciplinary Research Team

The consensual review and validation were carried out with the involvement of representatives of the main beneficiaries (older adults with and without cognitive decline) and of the research team, which is itself multidisciplinary and includes researchers in gerontology and ageing, neuropsychology, oral literature and ethnography, ethnomusicology/traditional dance, and computer science/artificial intelligence. The team evaluated and compared the different exercises, considering not only the cultural domains but also the dimensions of directional, semantic, idiomatic and conceptual equivalence of the content of each task. If a task was determined not to be applicable, it was modified to be more applicable to people with a neurocognitive disorder.

Items were reviewed and selected by consensus during regular project meetings; items considered culturally, linguistically or developmentally inadequate were modified or excluded by the team. A formal Delphi procedure with quantified agreement scores was not implemented in this pilot, given its exploratory and feasibility-oriented scope. The development of a structured Delphi protocol with explicit inter-expert agreement thresholds, an external panel of peers and the corresponding quantitative consensus indicators is foreseen for the next phase of the project, in preparation for the planned randomised trial.

#### Intangible Heritage: Criteria for Inclusion in the App

All material to be included in the App database was selected against three explicit criteria:

- availability in databases with a Creative Commons licence, to facilitate access to other researchers wishing to continue the study;
- identical categories and genres of Intangible Cultural Heritage (ICH) for the Spanish and Portuguese versions, to allow comparison of the data collected;
- a documented link between the material and the participants’ regions of birth and life experience in both countries, so that the items could function as triggers for recall in RT sessions.

This last criterion was operationalised as follows: each candidate item was characterised by its (a) region of origin (NUTS-III level), (b) usual context of transmission (maternal care, work, festivity, leisure) and (c) approximate period of greatest circulation. An item was considered a candidate stimulus for a given participant only when the intersection of (a)–(c) overlapped with the participant’s biographical map (region of birth, profession of upbringing, age cohort), reconstructed in a brief life-story interview at recruitment. The resulting participant-specific set of items was then used as the input pool for the App personalisation algorithm.

#### Intangible Heritage Available for Integration into the App

When consulting the databases available in Spain (and in Catalonia) and in Portugal, we found that:

- the Catalan and Portuguese databases differ markedly in the number of entries, the type of material available and the cataloguing system; it was therefore not possible to subcategorise the different genres of the traditional songbook included in the “songbook” category of the App;
- some of the Portuguese songbook databases provide audio only, since at the time of their creation no technology was available for collecting and preserving the moving image;
- for the region of one of the Portuguese Day Care Centres no database existed that could be integrated into the App; therefore, the team had to collect and record on site all the material used in RT sessions with the participants of that centre;
- no nursery-song databases were available for Portuguese, Catalan or Spanish; all the nursery songs included in the App database had therefore to be recorded by the project;
- there is no consolidated, peer-validated database of traditional Iberian dances; the project *The Portuguese Dance Liking Itself* is still under development (http://adancaportuguesaagostardelapropria.pedexumbo.com/). For Catalan dances it was necessary to use a different type of database.

### Categorisation and Specificities of the Collection Selected for the App

**Fig. 1:**
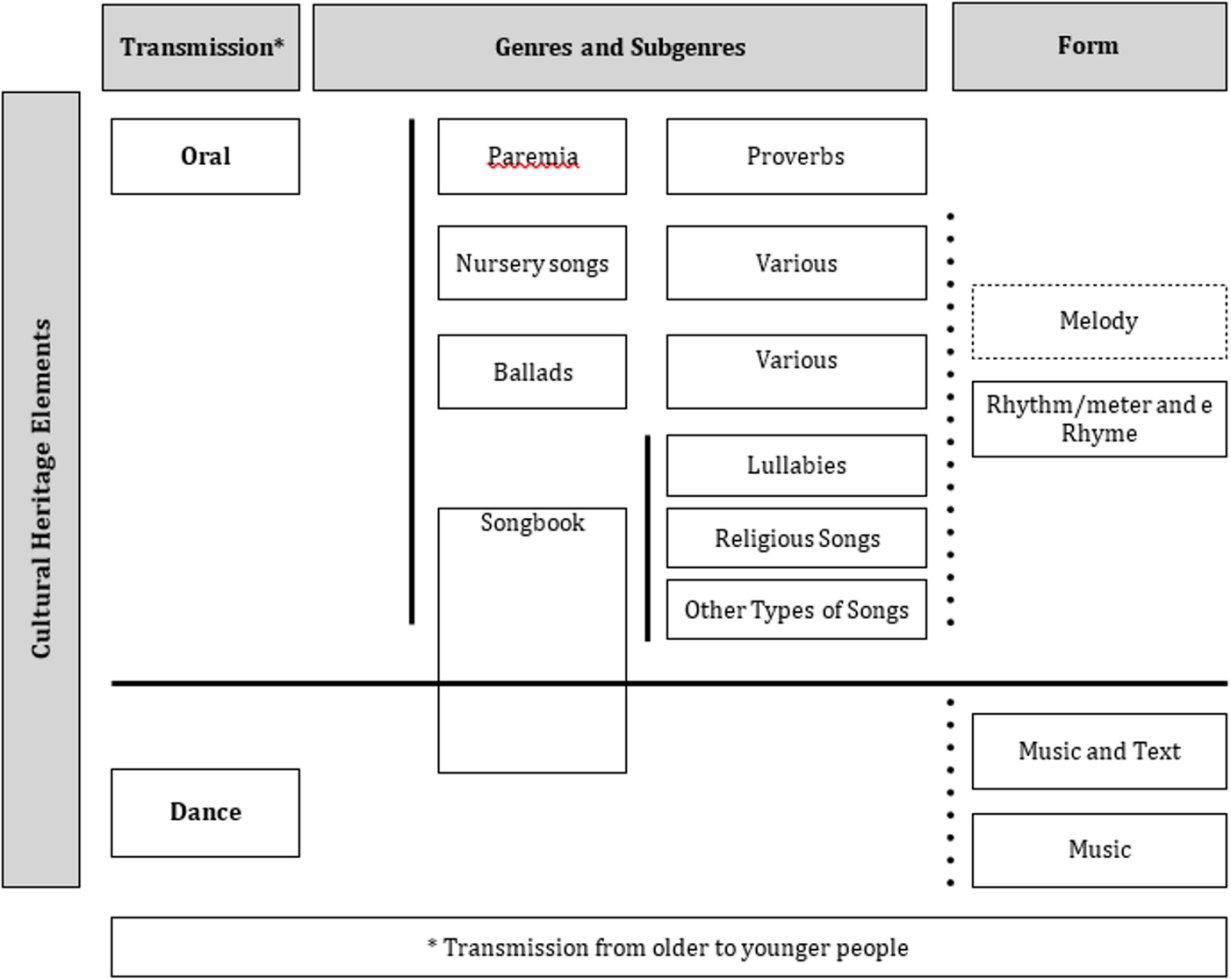
Categories of the ICH collection included in the App

Figure 1 below provides a synthetic overview of the categories of the ICH collection included in the App.

**Fig. 2:**
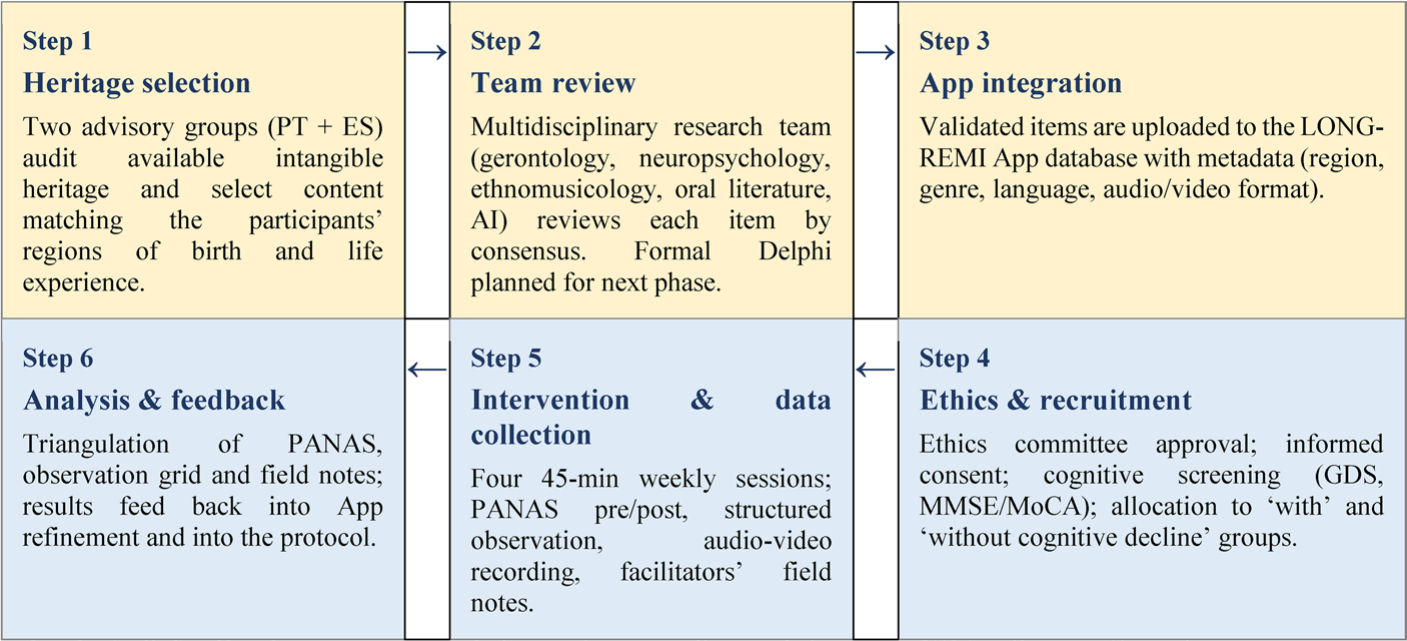
Workflow of the LONG-REMI study (heritage → analysis)

Table 2 below provides a synthetic overview of the methodological design of this study. Figure 2 schematises the workflow connecting heritage selection, expert validation, App integration, ethics and recruitment, intervention and analysis.

Among the ICH collection (*UNESCO – Intangible Cultural Heritage*, n.d.), only two types of heritage manifestations were included whose process of transmission (from older to younger generations) arises from specific contexts of intergenerational socialisation: dance and oral literature.

The dances chosen include dances whose performance can be accompanied either by melody alone or by a song with sung lyrics. From the collection of traditional literature (García Berrio & Huerta Calvo, 1992), textual typologies were selected whose function is related to (i) the acquisition and development of language and/or (ii) the reinforcement of identity and social regulation, in different contexts of socialisation: maternal care, work activity and festivity.

The chosen textual typologies have formal specificities that establish a relationship with mnemonic and reminiscence processes (Hayes, 2009; Santo, 2017). Closed textual genres were chosen which: (i) impose restrictions on the occurrence of variants; (ii) admit lexical variation; (iii) do not admit syntactic or fable variation (Catalán, 1997); (iv) almost always require the presence of rhyme; and (v) have an internal or external metric structure.

The corpus of traditional literature included in the App database is therefore made up of:

- nursery songs, ballads and songbook items, which are rhymed, long and/or brief genres, generally set to music but which also allow recitation without music;
- proverbs (a type of paremia), which are very brief textual forms, of anonymous origin, structured in two parts and which express popular wisdom regarding an ethical-moral theme or a worldview. From the collection of proverbs used by the Iberian tradition, only those with direct correspondence between Spanish, Catalan and Portuguese were selected.

## The Impact of Heritage Elements On Reminiscence Processes of People With and Without Cognitive Decline

The available literature considers that the metre of verse, rhyme and music function as triggers for language and emotion and, therefore, for memory (Belfi & Jakubowski, 2021; Bigand & Tillmann, 2020; Bower & Bolton, 1969; Hayes, 2009; Jakubowski et al., 2021; Ongchoco et al., 2022; Sacks, 2007). In the present pilot, 56 participants completed an average of 3.59 ± 0.40 of the four planned sessions. Usability and satisfaction were highly perceived in both groups (VAS usability 7.75 ± 1.88; VAS satisfaction 8.38 ± 1.57 on a 1–10 scale; CSUQ overall score 6.95 ± 0.22 in the group without cognitive decline and 5.44 ± 1.09 in the group with cognitive decline, on a 1–7 Likert). Pre/post-intervention scores on the PANAS positive affect subscale showed a statistically significant improvement (28.86 ± 8.88 vs. 36.70 ± 9.43; Z = − 4.18; *p* < 0.001), while no significant change was observed in the negative affect subscale (12.09 ± 3.19 vs. 11.89 ± 3.00; *p* = 0.688). The pattern was consistent in both groups (without cognitive decline: positive affect 31.81 ± 8.32 → 40.52 ± 10.17, *p* = 0.001; with cognitive decline: 27.09 ± 8.85 → 34.40 ± 8.28, *p* < 0.001). We observed that stimuli that combine rhyme and music (such as songbook items and ballads) produced the greatest recall of information and the strongest effect on communication and interaction with participants. We recorded moments in which participants spontaneously recalled other texts set to music, profane or religious. There were also occasions when ballads not set to music generated memories and influenced communication and interaction with participants, with episodes of ballad recitation being observed. Conversely, brief stimuli without music – nursery songs and proverbs – did not evoke memories in the participants.

As nursery songs are a rhythmic and rhyming type of text, used in contexts of intergenerational socialisation throughout life, the observed result is not in line with the literature, since one would expect their internal structure to evoke memories (Bower & Bolton, 1969; Connell, 2019; Jackendoff & Audring, 2020; Rainey & Larsen, 2002). At this stage of the investigation, it is not possible to determine whether the absence of recall was because the audio of these items was not supported by video, or to another factor (Table 3).

**Table 3:** Usability, satisfaction and PANAS scores by group (extracted from Doménech et al., 2023)

Outcome	Without cognitive decline (*n* = 21)	With cognitive decline (*n* = 35)
VAS Usability (1–10), M ± SD	8.29 ± 1.59	7.22 ± 2.18
VAS Satisfaction (1–10), M ± SD	8.57 ± 1.78	8.19 ± 1.36
CSUQ overall (1–7), M ± SD	6.95 ± 0.22	5.44 ± 1.09
PANAS Positive — pre, M ± SD	31.81 ± 8.32	27.09 ± 8.85
PANAS Positive — post, M ± SD	40.52 ± 10.17	34.40 ± 8.28
PANAS Positive — Wilcoxon Z; p	Z = − 3.18; *p* = 0.001	Z = − 3.60; *p* < 0.001
PANAS Negative — pre, M ± SD	11.76 ± 2.32	12.40 ± 3.62
PANAS Negative — post, M ± SD	11.62 ± 2.54	12.06 ± 3.26
PANAS Negative — Wilcoxon Z; p	Z = − 0.29; *p* = 0.775	Z = − 1.22; *p* = 0.224

Regarding proverbs, we are unaware of studies on their use as a stimulus for evoking memory. We therefore consider that more extensive studies are necessary to seek evidence of their relationship with mnemonic processes.

Throughout the sessions we also found that dances accompanied by music and lyrics produced more recall than dances accompanied only by music. During the sessions it was possible to record on video the foot movements of several participants under the table, following the dance steps shown on the screen.

According to the literature, the use of video in RT acts as a multisensory stimulus, since it combines vision and hearing. Videos, by being closer to reality and by increasing linguistic stimulation and memory, make more memories accessible (Belfi & Jakubowski, 2021; Donald, 2020; Janssen et al., 2007; Manav & Simsek, 2019). In our sessions, we observed that stimuli presented in video format produced more memory evocations than stimuli of which only the audio was presented. Nursery songs, proverbs and all the songs not accompanied by video generated less recall, less communication and less interaction, with episodes of disinterest being observed when participants were faced with a motionless screen.

Together, these observations suggest that the most effective stimuli, in our pilot, are those that combine three features simultaneously: rhyme and metre (textual structure), music (auditory dimension) and video (visual dimension). This pattern was consistent in both groups (with and without cognitive decline), although recall episodes were more frequent and more elaborate in participants without cognitive decline. These exploratory findings will need to be confirmed in a randomised, longitudinal trial with a larger sample.

## Conclusions

This pilot, mixed-methods, multicentre study – combining a methodological proposal with empirical observation – demonstrates that two specific manifestations of intangible heritage, traditional dance and traditional oral literature, can function as effective stimuli to evoke autobiographical memories in older adults with and without cognitive decline, when delivered through an AI-supported tablet App. More specifically, the use of the LONG-REMI App had observable effects on communication and interaction with people with and without cognitive decline. In a sample of 56 older adults (21 without cognitive decline; 35 with mild or moderate cognitive decline) recruited in six institutions in Spain and Portugal, the App was perceived as highly usable (VAS 7.75 ± 1.88) and satisfactory (VAS 8.38 ± 1.57), and produced a statistically significant increase in positive affect (PANAS positive subscale: 28.86 ± 8.88 → 36.70 ± 9.43; Z = − 4.18; *p* < 0.001) without an increase in negative affect. The study contributes three concrete outputs: (a) a reproducible protocol for cross-cultural selection and validation of intangible-heritage stimuli; (b) preliminary evidence on which formats (rhyme + music + video) maximise recall and engagement; and (c) an open methodological framework – including operational definitions of memory recall, verbal and non-verbal interaction, and a structured observation grid – that can be reused in future studies.

Limitations include the convenience sampling, the small sample size, the absence of a control group and the relatively short duration of the intervention (four sessions). It would therefore be important to increase the number of participants and carry out a randomised, longitudinal study with standardised instruments to validate these direct observations.

## Data Availability

The data that support the findings of this study are available from the corresponding author upon reasonable request.
